# The Clinical Utility of Post-Transplant Monitoring of Donor-Specific Antibodies in Stable Renal Transplant Recipients: A Consensus Report With Guideline Statements for Clinical Practice

**DOI:** 10.3389/ti.2023.11321

**Published:** 2023-07-25

**Authors:** Dennis A. J. van den Broek, Soufian Meziyerh, Klemens Budde, Carmen Lefaucheur, Emanuele Cozzi, Dominique Bertrand, Covadonga López del Moral, Anthony Dorling, Marie-Paule Emonds, Maarten Naesens, Aiko P. J. de Vries, the ESOT Working Group Subclinical DSA Monitoring

**Affiliations:** ^1^ Division of Nephrology, Department of Medicine, Leiden Transplant Center, Leiden University Medical Center, Leiden University, Leiden, Netherlands; ^2^ Department of Nephrology and Medical Intensive Care, Charité Universitätsmedizin Berlin, Berlin, Germany; ^3^ Paris Translational Research Center for Organ Transplantation, Kidney Transplant Department, Saint Louis Hospital, Université de Paris Cité, Paris, France; ^4^ Department of Cardiac, Thoracic and Vascular Sciences and Public Health, Transplant Immunology Unit, Padua University Hospital, Padua, Italy; ^5^ Department of Nephrology, Transplantation and Hemodialysis, Rouen University Hospital, Rouen, France; ^6^ Valdecilla Biomedical Research Institute (IDIVAL), Santander, Spain; ^7^ Department of Inflammation Biology, Centre for Nephrology, Urology and Transplantation, School of Immunology & Microbial Sciences, King’s College London, Guy’s Hospital, London, United Kingdom; ^8^ Histocompatibility and Immunogenetics Laboratory (HILA), Belgian Red Cross-Flanders, Mechelen, Belgium; ^9^ Department of Microbiology, Immunology and Transplantation, KU Leuven, Leuven, Belgium

**Keywords:** DSA, donor-specific HLA antibodies, biomarker, guidelines, subclinical rejection, monitoring

## Abstract

Solid phase immunoassays improved the detection and determination of the antigen-specificity of donor-specific antibodies (DSA) to human leukocyte antigens (HLA). The widespread use of SPI in kidney transplantation also introduced new clinical dilemmas, such as whether patients should be monitored for DSA pre- or post-transplantation. Pretransplant screening through SPI has become standard practice and DSA are readily determined in case of suspected rejection. However, DSA monitoring in recipients with stable graft function has not been universally established as standard of care. This may be related to uncertainty regarding the clinical utility of DSA monitoring as a screening tool. This consensus report aims to appraise the clinical utility of DSA monitoring in recipients without overt signs of graft dysfunction, using the Wilson & Junger criteria for assessing the validity of a screening practice. To assess the evidence on DSA monitoring, the European Society for Organ Transplantation (ESOT) convened a dedicated workgroup, comprised of experts in transplantation nephrology and immunology, to review relevant literature. Guidelines and statements were developed during a consensus conference by Delphi methodology that took place in person in November 2022 in Prague. The findings and recommendations of the workgroup on subclinical DSA monitoring are presented in this article.

## Introduction

The introduction of the complement-dependent cytotoxicity assay (CDC) in 1969 was the first step towards addressing the deleterious consequences of the humoral immune response and antibody-mediated rejection (ABMR) [1]. Means to investigate these entities were further expanded in later years by the introduction of novel techniques, amongst others, flow-cytometry and solid phase immunoassays (SPI). The use of the sensitive SPI also introduced new dilemmas, such as how to interpret SPI results in case of a negative pretransplant CDC-crossmatch or whether patients should be monitored for the incidence of donor-specific antibodies (DSA) to human leukocyte antigens (HLA) post-transplantation. A consensus meeting in 2013 concluded that pretransplant screening for potential DSA via single-antigen bead (SAB) SPI could be of benefit in risk stratification [2]. As a result, organ allocation organizations have mandated pretransplant screening of HLA antibodies through SAB-SPI as immunological risk stratification in order to define non-acceptable HLA antigens [3]. A recent position paper on pretransplant immunologic risk stratification adds further arguments for this screening practice [4]. Post-transplant monitoring of DSA in patients with graft dysfunction seems to be equally standard practice in case of clinical suspicion of ABMR [5, 6]. However, standardized monitoring of DSA in kidney transplant recipients (KTR) without signs of overt transplant dysfunction, so called *subclinical DSA*, has not universally taken hold as standard of care in most transplant centers. This is likely related to uncertainty regarding the clinical utility of standardized monitoring for subclinical DSA. The main aim of subclinical DSA monitoring is to identify patients who are at greater risk for rejection, either incipient or future, which makes it a form of (transplant) population screening. For such a strategy to have clinical utility, diagnostic and therapeutic ramifications need to be defined in case a patient is identified through screening and these consequences should lead to improved graft and patient outcomes. This may relate to earlier diagnosis and treatment of underlying subclinical rejection, but perhaps also to adaptation of maintenance treatment strategies to prevent future rejection. Additionally, cost-effectiveness of such practices should be considered. While DSA monitoring in stable patients has been recommended in previous guidelines, potential benefits of its consequences were largely unknown, especially in regards to treatment of underlying subclinical rejection [2]. This could possibly explain why some centers were hesitant to implement such strategies. However, uncertainties regarding effective therapeutic ramifications may counteract potential benefits of early detection. This limits potential further improvements in long-term allograft survival from an ontological reductionist view on alloimmunity. In the wake of new developments in this field over the past decade, this consensus report aims to appraise the clinical utility of regular standardized post-transplant monitoring of DSA in stable KTR. We will utilize the criteria for successful screening as developed by Wilson & Jungner in 1964, to ensure that all relevant aspects are reviewed [7] (Table 1). Additionally, potential knowledge gaps are identified and future research objectives stated.

**TABLE 1:** Wilson & Jungner’s principles of screening.

1. The condition sought should be an important health problem
2. The natural history of the condition, including development from latent to declared disease, should be adequately understood
3. There should be a recognizable latent or early symptomatic stage
4. There should be a suitable test or examination
5. The test should be acceptable to the population
6. There should be an agreed policy on whom to treat as patients
7. There should be an accepted treatment for patients with recognized disease
8. Facilities for diagnosis and treatment should be available
9. The cost of case-finding (including diagnosis and treatment of patients diagnosed) should be economically balanced in relation to possible expenditure on medical care as a whole
10. Case-finding should be a continuing process and not a “once and for all” project

To formulate this consensus statement, the European Society for Organ Transplantation (ESOT) convened a consensus conference, comprised of a European panel of experts in transplantation nephrology and immunology. The aim of this conference was to develop guidelines on DSA monitoring. The panel and juries were presented with summaries of evidence. Consensus statements and recommendations, and the Wilson & Jungner criteria they reflect, are summarized in Table 2. This document, which will be updated to reflect new evidence as it becomes available, is intended for healthcare providers.

**TABLE 2:** Summary of statements and recommendations.

Recommendations	GRADE level	W&J criterium
Efforts should be made to prevent late renal allograft loss, of which one of the leading causes is ABMR.	1A	1
Clinicians should note that DSA are associated with a high risk of rejection, primarily ABMR, and subsequent allograft loss	1A	2
DSA can signal for underlying microscopic injury, indicative of subclinical rejection (ABMR and TCMR), which can be identified through allograft biopsy	1C	3
Upon detection of *de novo* DSA, the pathogenicity and the impact on prognosis is currently best assessed by doing a biopsy	1C	3
Efforts should be made to standardize testing and reporting of DSA, including information on MFI, their plausibility and possible cross-reactive antigens/epitopes	1B	4,5,8
Whilst post-transplant monitoring of preformed DSA in patients with stable graft function might be helpful, additional clinical and laboratory parameters should also be considered when deciding if a biopsy should be performed	2C	4,5,8
DSA MFI levels or complement binding ability (C1q, C4d, C3d) should not influence decision-making regarding whether a biopsy in patients with subclinical dnDSA should be performed	2C	4,5,8
We recommend optimization of maintenance therapy, including addressing non-adherence, in patients who develop subclinical dnDSA. Additional treatment should only be considered after performing an allograft biopsy	1C	6–7
Cost-effectiveness of DSA monitoring in patients with stable graft function depends on incidence rate of dnDSA and importantly on size effect of treatment	2D	9
Monitoring for dnDSA during functional graft life is a continuous process and should not change upon detection of dnDSA.	2C	10
The optimal DSA monitoring scheme has not been established, but a routine approach would be antibody monitoring at three to six months post-transplant and annually thereafter	2C	10

## Methods

The consensus development process was organized by a dedicated Guidelines Taskforce within ESOT and its sections ELITA, EKITA, EPITA, ECTTA, ETHAP, Education Committee, YPT, Transplant International editorial board members and patient representatives. The detailed description of methodology used is reported previously [8]. Briefly, key issues were identified by each workgroup and specific clinical questions were formulated according to the PICO methodology (PICO = Population, Intervention, Comparator and Outcome). All PICO questions are listed in Table 3. Following the definition of the PICOs, literature searches were developed by expert staff from the Centre for Evidence in Transplantation, who have expertise in conducting systematic reviews and subsequently integrated, when needed, by the steering committee experts. The workgroup proposed a recommendation for each key question, based on the quality of evidence rated using the GRADE approach, with high quality rated as A, medium quality as B, and low quality as C; very low quality of evidence was not considered. For evaluation of the quality of evidence according to GRADE the following features were considered: study design, risk of bias, inconsistency, indirectness, imprecision, number of patients, effect, importance and publication bias. Strength of recommendation was rated as 1 (strong) or 2 (weak).

**TABLE 3:** Overarching questions & PICOs.

W&J criterium	Overarching question	PICO(s)	
1	Does late rejection pose a health problem?	In renal transplant recipients (P), is late rejection (I) a significant contributor to allograft attrition rates compared to other factors (C)?	
2	Do we understand the natural history of rejection sufficiently?	In renal transplant recipients with rejection (P), are DSA (I) a significant independent causative contributor to development of the rejection process (O) compared to those without DSA (C)?	
		In renal transplant recipient with rejection (P), are other factors (I) determined as significant independent cause for the development of the rejection process (O) compared to those without those factors (C)?	
3	Are we able to identify latent rejection through DSA screening before overt dysfunction occurs?	In renal transplant recipients (P), is development of dnDSA or prevalence of preformed DSA (I) associated with subclinical rejection (O) compared to those without DSA (C)?	
		In renal transplant recipients with subclinical DSA (P), can allograft biopsy guided by DSA development/evolution (I) identify subclinical rejection in an earlier pathological stage (O) compared to biopsies in the event of more overt dysfunction (C)?	
4,5,8	Are current DSA testing methods suitable for DSA screening and can certain DSA characteristics be used to further guide allograft biopsy decision making	In renal transplant recipients are current DSA assessment methods sufficient to reliably detect anti-HLA antibodies and their donor specificity?	
		In renal transplant recipients with subclinical DSA (P), can DSA characteristics (MFI, class, IgG subclass, complement binding ability) (I), reliably be used to identify patients without rejection (O) compared to allograft biopsy (C)?	
6–7	Is treatment for patients with subclinical DSA or subclinical rejection defined?	In renal transplant recipients with subclinical DSA who have not yet been biopsied (P), is treatment of any kind (I) compared to no treatment (C) beneficial for transplant outcome (O) (allograft loss, clinical rejection risk)?
		In renal transplant recipients with rejection (ABMR or TCMR) (P), is treatment in the subclinical phase (I) more beneficial to transplant outcome (O) (allograft loss/kidney function) compared to treatment in case of overt dysfunction (C)?
9	Is there any evidence of cost-effectiveness of standardized DSA monitoring and treatment of found cases?	In renal transplant recipients (P), has monitoring of DSA (I) been shown to be cost-effective compared to no monitoring of DSA (C)?
10	How frequent and until what time should DSA monitoring be conducted?	Is the incidence rate as a function of time post-transplant defined?
		In renal transplant recipients who have developed dnDSA (P), is development of additional dnDSA (I) associated with worse transplant outcome (O), compared to no additional dnDSA (C)?
		In renal transplant recipients who have developed dnDSA (P), is disappearance of the dnDSA (I) associated with better transplant outcomes (O) compared to persistence (C)?
		In renal transplant recipients (P), are clear risk categories (I) defined for the risk of development of dnDSA (O) compared to those without those risks (C)?
		In renal transplant recipients (P), are certain monitoring frequencies (annually, biannually, etc.) (I) associated with better transplant outcomes (O) compared to other monitoring frequencies (C)?

The Delphi method was applied to arrive at a group opinion during the consensus conference. Complete information, including the list of consensus conference workgroup domains and topics, and the process regarding consensus conference participant selection, development and refinement of consensus statements, and modified Delphi methodology including consensus polling, were determined before the conference held in Prague, Czech Republic, November 13–15, 2022, as previously reported [8] Table 3.

### Efforts Should Be Made to Prevent Late Renal Allograft Loss, of Which One of the Leading Causes Is ABMR. (1A)

For a successful screening strategy it is important that the disease is relevant and constitutes an significant health problem. Breakthroughs in maintenance immunosuppression during the latter part of the past century drastically increased kidney graft survival rates [9]. This was, however, realized mainly through increases in graft survival over the first year. Comparably less progress has been made in improving graft attrition rates beyond the first year during this era. However, more recent European analysis of collaborative transplant study (CTS) data showed that improvement of long-term graft survival since 2000 was greater than short-term advancement, independently of changing donor and recipient characteristics, likely reflecting the evolutions in posttransplant monitoring and management [10, 11]. An important limiting factor to prolong long-term death-censored graft survival is the development of antibody-mediated rejection (ABMR), in which DSA play an important role [12]. This entity is recognized as a major cause for overall death-censored renal allograft loss in recent decades [13, 14]. The Banff ’19 pathology classification recognizes three forms of ABMR in renal allografts: active, chronic active and chronic ABMR [5]. Even though there is incidental empiric evidence for reversal in the case of (hyper)active forms of ABMR [15], all forms of ABMR infer a great risk for graft failure [16]. A recent analysis attributed around a third of all allograft loss to ABMR, particularly contributing to late allograft failure [17]. Therefore, it seems undisputable that diminishing the rate of allograft loss due to ABMR is an important health issue in kidney transplantation and we recommend that efforts to further improve long-term graft survival should explore new openings to steer away from the current diagnostic and therapeutic nihilistic view on chronic rejection.

### Clinicians Should Note That DSA Are Associated With a High Risk of Rejection, Primarily ABMR, and Subsequent Allograft Loss. (1A)

#### Epidemiological Associations Between HLA-DSA and Allograft Outcome

For screening to be successful, one should have an understanding of how underlying pathological processes can develop into overt graft dysfunction.

In case of ABMR, the screening marker itself seems to be implicated in the underlying pathological process [6, 12]. This is apparent with pretransplant DSA, considering the high risk of hyperacute rejection if transplantation proceeds despite a positive CDC-crossmatch. Modern practice precludes such transplantation with pretransplant listing of non-acceptable HLA antigens, or with measures such as paired kidney exchange programs or desensitization in the living donation setting. In contrast, CDC-crossmatch negative pretransplant DSA, which are identified through SPI only, are not necessarily a contraindication to transplantation in patients faced with no alternatives beyond dialysis [3]. However, these DSA still convey increased risk for ABMR and allograft loss according to a meta-analysis by Mohan et al. [18] Recent analysis of CTS data indicated that nearly 15% of recipients of deceased donor kidneys with crossmatch negative pretransplant DSA progressed to allograft failure within the first year post-transplant [19]. This figure was even higher in retransplant patients. In regards to dnDSA, a large meta-analysis by Sharma et al. [20] implicated development of dnDSA as a severe risk factor for notably cellular rejection, acute ABMR (aABMR), chronic ABMR (cABMR), and allograft loss. Moreover, CTS data showed that 20% of patients who developed dnDSA in the first post-transplant year progressed to allograft failure within the next five [19]. A recent randomized trial corroborated these results [21].

#### Pathogenesis of HLA-DSA and Plausible Causality With Subsequent Rejection

The genesis of DSA after transplantation is a complex process. B-cells can initiate and subsequently differentiated plasma cells (as well as B-cells) can maintain production of these antibodies as a result of sensitization of the adaptive immune system. Sensitization could be related to a period of underexposure, either due to non-adherence or iatrogenic reduction of immunosuppression [22–28]. Additionally, poor HLA matching [28–31] and previous episodes of T-cell mediated rejection (TCMR) [29, 30, 32–34] have been associated with DSA development. Other risk factors pertain to certain recipient characteristics such as age or ethnicity [35, 36]. The association of previous TCMR and dnDSA development is hypothesized to be explained by sensitization of the B-cell compartment through inflammation induced by T-cell alloimmunity, especially T-follicular helper cells [37–39]. The role of T-cells in a process which could ultimately lead to ABMR seems to question the dichotomous view on rejection (i.e., either TCMR or ABMR as separate entities). Perhaps a more contemporary view on rejection is that it is a heterogenous spectrum with different histological and clinical manifestations [40].

While the process of sensitization leading to DSA formation is complex and multifactorial, the risks DSA convey are clear. Still, this does not necessarily infer a causal relationship. Though the pathogenicity of HLA-DSA was extensively studied in recent years and a recent thorough literature review by Callemeyn et al. [40] attempted to untangle association from causation. This review assessed the possible causal relationship between HLA-DSA and microvascular inflammation (MVI), a histopathological hallmark of ABMR, through the Bradford-Hill criteria, which can be used as guide for causal inference in epidemiological research. These criteria include: strength of effect size and reproducibility, experimental evidence *in vitro* and *in vivo*, temporality between HLA-DSA appearance and graft injury, biological gradient, and coherence and analogy [40, 41]. Callemeyn et al; [40] illustrates that most criteria are met. However, more investigations are warranted to demonstrate a clear biological gradient between antibody titre and occurrence of ABMR or graft failure; [42]. Yet, recent studies by Viglietti et al; [43, 44] showed that treatment of ABMR through plasmapheresis (PP), intravenous immunoglobulins (IVIG) and rituximab is associated with a significant decrease in DSA MFI and capacity to bind C1q. Interestingly, these reductions in DSA properties were significantly associated with improved graft survival in patients with ABMR. However, treatment effects on more chronic or late ABMR are variable [15]. Furthermore, the histological presentation of ABMR including MVI is not always specific for antibody involvement, as other causes could appear clinicopathologically similar. Nonetheless, there seems to be clear preclinical and clinical evidence of a pathogenic relation between HLA-DSA and ABMR.

#### Mechanisms of HLA-DSA-Induced Allograft Damage to Explain Phenotypic Variability

Despite this strong relationship, not all recipients with preformed DSA or dnDSA seem to progress to ABMR or graft failure [16, 29, 45]. Multiple mechanisms have been proposed to explain this variation in effect of HLA-DSA on graft outcomes. A recent comprehensive review has summarized HLA-DSA attributes and discussed mechanisms of HLA-DSA-induced effector functions in mediating allograft damage [46]. These effector functions may be Fc-dependent, such as the impact of antibody glycosylation status on complement activation and recruitment of cytotoxic NK-cells and macrophages [47, 48]. Regarding Fc-independent mechanisms, recent studies describe intracellular signalling downstream of HLA-antibody binding to endothelial cells that promote upregulation of adhesion molecules, proliferation and activation of endothelial cells, induction of dendritic cells and CD4^+^ T-cell maturation [46, 49, 50]. HLA-antibody ligation of the HLA-molecule of endothelial cells can also lead to anaphylatoxin production that can result in more monocyte recruitment. Recruitment is also mediated by the cellular expression of anaphylatoxin receptors on CD4^+^ and CD8^+^ T cells, and myeloid cells [51, 52]. Lastly, regulatory T and B-cell populations may play a pivotal role in suppressing the deleterious effects of DSA on the graft. Recent research indicates that these cell lines impart tolerogenic effects through impairment of the T-follicular helper cell – B-cell interaction and that these regulatory cells were significantly reduced in frequency in patients with DSA who developed ABMR, as compared to patients with DSA but absent ABMR [38, 39, 53].

#### Relationship Between HLA-DSA Properties and Allograft Injury Phenotypes

Several studies have shown that high titre HLA-DSA reflected by high MFI levels, and inflammatory isotype switching toward IgG1 and IgG3, and thus their capacity to bind C1q or C3d are associated with significantly increased microvascular inflammation and C4d deposition [54, 55]. Although considered classically non-inflammatory, the IgG4 isotype has been associated with subclinical graft rejection, including ABMR, in several studies in kidney and other solid organ transplants [55, 56]. Subclinical ABMR was shown to lead to significantly more transplant glomerulopathy and accelerated graft loss when compared to subclinical TCMR [57]. In addition to subclinical ABMR, HLA-DSA have been shown to be significantly associated with kidney graft fibrosis and subsequent accelerated graft loss [58]. The relationship between HLA-DSA and graft fibrosis was independent of previous ABMR episodes. Thus, HLA-DSA, even detected at low strength/MFI, are associated with subclinical damage and fibrosis independent of clinical ABMR occurrence [58, 59].

#### HLA-DSA Independent Mechanisms of Microvascular Inflammation

Lastly, it must be mentioned that not all patients with MVI, indicative of injury attributed as being “antibody-mediated” by current Banff criteria, have detectable levels of HLA-DSA. The histopathologic entity of MVI without detectable HLA-DSA by definition suggests that factors other than HLA-DSA may mediate MVI, such as non-HLA antibodies [60–63]. Antibody-*in*dependent pathways may include NK-cell alloimmunity through a “missing-self” mechanism [64, 65] or direct allorecognition by monocytes [66, 67]. Other causes may not even be related directly to alloimmunity, such as recurrent complement-mediated renal disease, ischemia/reperfusion injury, or viral endothelial infection.

The above presented body of evidence illustrates that there are likely multiple individual pathways, not all of which are fully understood, that eventually lead to varying levels of microscopic injury that is currently defined as MVI and ABMR, which may need some clarification [6, 68]. Nonetheless, regardless of the incompletely understood natural history of ABMR and MVI, there is a large amount of preclinical and clinical evidence warranting strong support for the notion that anti-HLA-DSA are significantly associated with, and predictive of rejection and clinicians should be aware of this [5, 12, 15, 40].

### DSA can Be a Signal for Underlying Microscopic Injury, Indicative of Subclinical Rejection (ABMR and TCMR), Which can Be Identified Through Allograft Biopsy. (1C)

#### Subclinical DSA as a Marker for Latent Rejection

For a valid screening strategy for disease, clinicians should also be able to identify a latent stage. In some cases, patients undergoing rejection present with clinical dysfunction of the graft as first sign. However, most have a latent phase with prevalent DSA prior to developing graft dysfunction. The first evidence of subclinical DSA as a marker for latent rejection came from preclinical studies in a non-human primate model with sequential protocol biopsies by Smith et al. [69, 70] They showed that development of dnDSA generally precedes graft dysfunction, as well as C4d-deposition or transplant glomerulopathy. Clinical studies of longitudinal protocol biopsies in stable renal transplant recipients with preformed DSA show substantial oscillations characterized by fluctuations in HLA-DSAs, C4d deposition and scores for glomerulitis and/or capillaritis in a dynamic and multidirectional fashion [71–73]. Seminal papers by Wiebe et al. [22, 34] have shown that this progressive subclinical injury can also be detected in patients with dnDSA several years after kidney transplantation. They found that of 64 patients who developed dnDSA, the majority was without graft dysfunction. Additionally, development of subclinical dnDSA was independently associated with transplant glomerulopathy (and thus chronic ABMR (cABMR)), decline in graft function, and allograft loss. Therefore, it is unlikely that chronic rejection is the result of a single spike of HLA-DSA or a single episode of ABMR. Instead, it represents a dynamic process that continues, unabated, at varying levels and eventually progresses towards chronic allograft injury, graft dysfunction and ultimately graft loss [12].

Development of latent rejection in patients with subclinical DSA has been observed in other types of organ transplants [74–76], as well as in more recent clinical studies in KTR, which show underlying rejection in roughly half of overall patients (Table 4). Bertrand et al. [77] recently analyzed 123 patients with subclinical dnDSA in a French multicenter cohort study and found that 41.5% of these patients had subclinical ABMR. Loupy et al. [57] showed in a large prospective cohort study of 1,001 patients with 1 year protocol biopsies that of 256 patients with subclinical DSA, 55% had ABMR. Of these cases, 78% were related to pretransplant DSA, further indicating that both pretransplant DSA and dnDSA can underlie a latent pathological process. Coemans et al. [78] recently studied longitudinal protocol and indication biopsies in a single-centre cohort of 1,000 Belgian patients. Of these, 108 had pretransplant DSA and 47 developed dnDSA. The prevalence of subclinical aABMR in protocol biopsies at 3, 12, 24 and 60 months post-transplant was 42.5%, 40.5%, 37.3% and 13.3% respectively in patients with HLA-DSA. Prevalence of transplant glomerulopathy increased over time and this was associated with previously diagnosed aABMR, further corroborating the notion that ABMR is a dynamic and continuous process [71, 72]. Schinstock et al. [30] retrospectively analyzed a single center cohort of patients with serial surveillance biopsies, but also included biopsies at graft dysfunction and upon subclinical dnDSA development. They found that of the 40 patients who were biopsied at the time of dnDSA development, 25%, 7.5% and 20% had underlying aABMR, cABMR, and TCMR respectively. Yamamoto et al. [79] reported on a Japanese cohort of 43 patients with subclinical dnDSA and found that 41.8% of patients had ABMR. Parajuli et al. [80] showed in an American retrospective single center cohort study with biopsies in case of dnDSA development or clinical indication that of 29 patients with subclinical dnDSA, 15 (51%) had underlying rejection. Of those rejections, 60% were ABMR, 20% mixed rejections, and 20% were TCMR. Waldecker et al. [81] retrospectively studied 84 German patients with indication biopsies or in case of dnDSA development from a single centre and found that out of 50 patients with subclinical dnDSA, 44% had ABMR, 15% had TCMR, 12% had mixed rejection and 15% had borderline rejection. Notably, only 14% of these patients had no histopathological signs of rejection at light microscopy. Eskandary et al. [82] retrospectively reported on the screening process for the BORTEJECT study, whereby 861 patients with stable grafts were cross-sectionally screened for presence of DSA [82]. Of 86 patients with subclinical DSA, 44 (51%) met the Banff criteria for ABMR. Lastly, Cornell et al. [83] analysed the results of a prospective trial on pretransplant desensitization with eculizumab in patients with a positive flow-crossmatch and compared the long-term outcomes to a historical matched cohort. The overall prevalence of subclinical ABMR at 3 months, 1 year and 2 years post-transplant was 41.8%, 37% and 20% respectively in a total of 78 patients.

**TABLE 4:** Summary of studies on subclinical DSA in renal transplant recipients.

Study	Type of study	Total patients (n)	Total DSA+	Biopsied patients with subclinical DSA (n)	dnDSA/preformed DSA	Time of biopsy	Subclinical aABMR (n) (%)*	Subclinical caABMR (n) (%)*	Subclinical cABMR (n) (%)*	Subclinical TCMR (n) (%)*	Subclinical mixed rejection (n) (%)*	No rejection (n) (%)*	Outcome
Wiebe et al. [22, 34]	Retrospective Single center	508	64	45	dnDSA	6 months post-transplant At dnDSA detection Graft dysfunction	Not specified	Not specified	Not specified	Not specified	Not specified	Not specified	Time to 50% allograft loss in clinical dnDSA vs. subclinical dnDSA: 3.3 years vs. 8.8 years (*p* < 0.0001)
													Significantly worse allograft survival in subclinical dnDSA vs. no dnDSA + no dysfunction
Bertrand et al. [77]	Retrospective Multicenter	123	123	123	dnDSA	At dnDSA detection	32 (26%)	19 (15.5%)	Not specified	Not specified	Not specified	No ABMR: 72 (58.5%)	Significantly worse post-biopsy 8-years allograft survival and 5 years delta creatinine in subclinical aABMR and cABMR compared to dnDSA without rejection
Loupy et al. [57]	Retrospective Single center + external validation	1,001	256	256	Preformed DSA + dnDSA	1 year post-transplant	With DSA: 142 (55%)*			With DSA: 17 (6.6%)*	Not specified	With DSA: 97 (38%)*	Significantly worse post-biopsy 8-years allograft survival and delta creatinine in subclinical ABMR compared to subclinical TCMR or no rejection
							Total: 142 (14.2%)**			Total: 132 (13.2%)**		Total: 727 (72.6%)**	No significant difference between (treated) subclinical TCMR and no rejection in either allograft survival or delta creatinine
Coemans et al. [78]	Retrospective Single center	1,000	155	At 3 months: 60	Preformed DSA (108) + dnDSA (47)	3, 12, and 24 months post-transplant	At 3 months: 42.5%	Not specified	Not specified	Not specified	Not specified	No aABMR at 3 months: 57.5%	No analysis of effect of subclinical rejection vs. no rejection on transplant outcome
				At 12 months: 37		Additional protocol biopsy at either 3, 4 or 5 years post-transplant	At 1 year: 40.5%					At 12 months: 59.5%	
				At 24 months: 29		Indication	At 2 years: 37.3%					At 24 months: 62.7%	
				At 60 months: 15			At 5 years: 13.3%					At 5 years: 86.7%	
Schinstock et al. [30]	Retrospective Single center	771	54	40 biopsied at detection of DSA	dnDSA	4, 12, 24, 60 months post-transplant at dnDSA detection Graft dysfunction	At dnDSA detection: 10 (25%)	Not specified	At dnDSA detection: 3 (7.5%)	At dnDSA detection: 8 (20%)	Not specified	Not specified	Only those with dnDSA + ABMR had evidence of graft loss at mean follow up of 3.2 ± 2.0 years
				34 biopsied 1 year post detection of DSA			1 year post dnDSA detection 18 (53%)		1 year post dnDSA detection 13 (38.2%)	1 year post dnDSA detection 5 (14.7%)			21.4% vs. 0% in dnDSA without AMR. (*p* < 0.01)
				Not all subclinical									No significant difference in composite endpoint of −50% eGFR or allograft loss between dnDSA without AMR vs. no dnDSA (*p* = 0.26)
Yamamoto et al. [79]	Retrospective Single center	899	95	43	dnDSA	At dnDSA detection	18 (42%) At rebiopsy 2 years post biopsy in those without ABMR: 0 (0%)			Not specified	Not specified	No ABMR: 25 (58%)	Only 1 of 11 patients at 2 years follow up without ABMR at initial biopsy had deteriorating creatinemia/proteinuria
												At rebiopsy 2 years post biopsy in those without ABMR: 8 (100%)	
Parajuli et al. [80]	Retrospective Single center	45	45	29	dnDSA	At dnDSA detection “Other indications"	9 (31%)			3 (10%)	3 (10%)	14 (48%)	Significantly better 1 year post biopsy eGFR in patients with subclinical dnDSA vs. clinical dnDSA.
													No statistical differences in allograft loss rate but low event rate
Waldecker et al. [81]	Retrospective Single center	865	132	34	dnDSA	At dnDSA detection Graft dysfunction	11 (26%)	3 (9%)	1 (3%)	5 (15%)	4 (12%)	5 (15%)	No analysis of effect of subclinical rejection on transplant outcomes
Eskandary et al. [82]	Retrospective Single center	861	86	86	Preformed DSA + dnDSA	Cross-sectional screening	44 (51%)			Not specified	Not specified	No ABMR 42 (49%)	Only patients with subclinical ABMR had evidence of graft loss
													5 vs. 0 without rejection during a median follow up of 20.5 months
Cornell et al. [83]	Prospective trial cohort + historical retrospective matched cohort	78	78	78	Preformed DSA	3–4, 12 and 24 months post-transplant	Overall: At 3–4 months: 41.8%			Not specified	Not specified	Overall: No ABMR at 3–4 months: 58.2%	No analysis of effect of subclinical rejection vs. no rejection on transplant outcome
							At 1 year: 37%					At 1 year: 63%	
							At 2 years: 20%					At 2 years: 80%	

ABMR, antibody-mediated rejection; aABMR, active ABMR; cABMR, Chronic ABMR; caABMR, Chronic active ABMR; DSA, Donor specific antibody; TCMR, T-cell mediated rejection. *: Proportion of total subclinical DSA patients with a biopsy **: Proportion of total patients.

#### Relationship Between *de novo* DSA and Subclinical T-cell Mediated Rejection

While most studies only reported these biopsy results in terms of either positive or negative for ABMR, the studies by Schinstock et al. [30], Parajuli et al. [80] and Waldecker et al. [81] interestingly further show that subclinical dnDSA can also be a signal for underlying TCMR. Unfortunately, no biopsies were performed in a DSA-negative control group in these studies, making it difficult to ascertain the precise odds of dnDSA to signal TCMR risk. Nonetheless, as discussed above, the association between TCMR and dnDSA development has been described previously in multiple studies. The study by Loupy et al. [57] seems to contrast this suggested association, as they showed that significantly more patients without DSA developed subclinical TCMR compared to patients with DSA. However, the majority of patients with DSA in this study had preformed DSA, not dnDSA. This was also reflected in patients with subclinical TCMR, as only 5 out of 17 (29%) of these patients had dnDSA. This could imply that there is no association between preformed DSA and TCMR, while there might be one for dnDSA and TCMR. Others were also not able to relate pretransplant DSA and TCMR development [84, 85]. However, these studies did not take into account the possible presence of mixed rejection in these analyses, or pooled these type of rejections with patients with ABMR. The study by Coemans et al. [78] did adjust their analysis accordingly and found that while pretransplant DSA was not associated with isolated TCMR, it was associated with total TCMR including mixed rejections. Nevertheless, the results of these studies might be further evidence for the previously described view on rejection as a spectrum of different clinical and histological manifestations. These may occur in sequence, as TCMR can result in dnDSA development, which can lead to ABMR. In contrast, preformed DSA may not necessarily lead to development of isolated TCMR. Collectively, these results imply that a biopsy serves to diagnose latent rejection (ABMR, TCMR or mixed rejection) in around half of patients with subclinical DSA, which is in line with previous recommendations on performing an allograft biopsy these patients [15]. While the number of papers warrants strong support for this statement, the evidence is mainly observational. This is reflected in the grading.

### Upon Detection of a dnDSA, the Pathogenicity and the Impact on Prognosis is Currently Best Assessed by Doing a Biopsy (1C)

#### Prognostic Value of a Banff Classified Rejection Diagnosis

Aside from the diagnostic purposes of an allograft biopsy in patients with subclinical DSA, it may also have prognostic value in predicting allograft loss. As stated before, not all patients who develop DSA seem to lose their graft or even show declining allograft function. Wiebe et al. [22] showed that 50% of patients still had a functioning allograft 10 years post-detection of the subclinical dnDSA. Therefore, further risk stratification regarding the pathogenicity of these subclinical dnDSA seems necessary. The Banff classified rejection diagnosis in these patients may provide further risk-stratification for detrimental transplant outcomes. Multiple studies showed that KTR with subclinical DSA and histological evidence of ABMR had significantly worse allograft survival rates and allograft functional decline than those without histopathological rejection at light microscopy [30, 57, 77, 78, 86]. Others show a similar trend, albeit not statistically significant [79, 82]. Moreover, Bertrand et al. [77] and Loupy et al. [57] showed that patients with subclinical dnDSA but without ABMR had excellent 8 years allograft survival (>90%) and stable graft function. This suggests that the rejection diagnosis could be prognostically more important for graft outcome than the dnDSA status itself in patients without graft dysfunction. This was further corroborated by Parajuli et al. [87] in a cohort of 587 patients without rejection at an initial protocol or indication biopsy, whereafter there was no difference in 5 years allograft loss rates between DSA-positive and DSA-negative patients. Though, dnDSA positivity in patients with a negative index biopsy was associated with subsequent rejection.

#### Prognostic Value of Inflammation Activity

Beyond the prognostic value of the Banff classified rejection diagnosis in patients with subclinical DSA, the severity of the Banff recognized acute pathological lesions may perhaps offer further risk-stratification in patients with rejection. A prospective cohort study of 215 patients showed that while DSA were univariately associated with renal function decline, this was no longer statistically significant when analyzed with a multivariate model including MVI and tubulitis [88]. This suggests that the presence of histological markers may define a more severe phenotype in patients with DSA. Wiebe et al. [22] showed in multivariate analysis of a small subcohort of 23 subclinical patients with dnDSA that tubulitis was a strong predictor for allograft loss. Studies by De Kort et al. [89] and the iBox study by Loupy et al. [90] elegantly showed that increasing levels of MVI severity score in patients with DSA is independently associated with worse allograft survival. A more recent study on semi-supervised clustering through data-driven mathematic modeling by Vaulet et al. [91] further corroborated the prognostic value of inflammation activity as determinant of allograft loss within patients with DSA.

#### Prognostic Value of Chronicity Markers

Histological chronicity markers also impact allograft survival. The presence of transplant glomerulopathy is implicated as independent risk factor for allograft attrition in multivariate analysis by multiple studies [22, 78, 90, 92]. Other chronicity markers such as interstitial fibrosis or tubular atrophy also seem to independently infer risk for allograft loss in patients with DSA [90]. More recent research showed that patients with increasing chronicity scores as determined by an aggregate of several chronicity markers have significantly worse prognosis in terms of allograft survival [93]. A follow-up study by Vaulet et al. [94] validated these results, again through a semi-supervised clustering approach, and found that clusters with higher levels of chronicity were associated with increasingly higher rates of allograft loss. Importantly, they assessed the impact of time since transplant of the biopsy in this study. Even though there was an association with clustering based on chronicity, clustering solely based on time of biopsy could not discriminate in allograft loss rate. This indicated that pattern of chronicity scores was an independent risk factor for poor allograft survival, regardless of the post-transplant time of the biopsy.

#### Temporal Association Between Activity and Chronicity and Relation With Efficacy of Treatment

Even though activity and chronicity are viewed as separate entities for the sake of the analyses in these studies, it should not be forgotten that they are intertwined. The temporal association between aABMR and chronic lesions associated with cABMR such as transplant glomerulopathy, peritubular capillary basement membrane multilayering and transplant arteriopathy is well described in both preclinical models and clinical studies [57, 69, 70, 78, 95–97]. Previous research in patients with TCMR has shown that chronic scarring is a determinate for poor response to treatment [98, 99]. Haas et al. [100] has also previously shown that early intervention in patients with ABMR may prevent chronic lesions such as transplant glomerulopathy. Recently, Wu et al. [101] did not observe any effect of current treatment options in patients with chronic ABMR and transplant glomerulopathy further indicating that late stages are less responsive to therapy. It could thus be of interest to identify patients at an earlier stage of the ABMR disease process. If a latent phase with subclinical DSA is an earlier stage in the continuum of rejection, then a biopsy taken at this stage may theoretically show less chronicity and these patients could perhaps be more amendable to treatment. Unfortunately, very few studies investigated this. Parajuli et al. [102] found that the Banff sum chronicity and transplant glomerulopathy scores of patients with underlying ABMR at biopsy were significantly lower in subclinical ABMR compared to dysfunctioning allografts with ABMR. Additionally, Wiebe et al. [34] found that no patients with subclinical dnDSA had evidence of transplant glomerulopathy. However, more research is needed to confirm this hypothesis, as current data is insufficient to draw meaningful conclusions.

#### Timing of the Allograft Biopsy

While this body of evidence seems to point out the additional prognostic value and possible clinical utility of an allograft biopsy in patients with (subclinical) DSA, it does not necessarily provide direction on when to perform this biopsy within the subclinical stage. The study by Schinstock et al. [30] clearly showed that within dnDSA-positive patients with a negative index biopsy, a follow-up biopsy 1 year later yields significantly more positive cases of ABMR. This appears to contrast aforementioned studies which suggested that an initial negative biopsy infers significantly less risk in patients who have developed dnDSA. However, the findings by Schinstock et al. [30] could be explained by the fact that not all their included biopsies at either dnDSA detection or follow-up were in fact in a subclinical setting. This may affect the *a priori* probability of finding underlying rejection at follow-up. Alternatively, the worse prognosis of the minority group who do eventually develop rejection may have been masked by the majority who remained without significant graft injury in other studies. Nevertheless, Schinstock et al. [30] could indicate that performing the biopsy too early could lead to false negative findings. Whereas no histopathological rejection at light or electron microscopy might be visible in these cases, there may in fact still be rejection at the molecular level. Previous research has shown the independent prognostic value of molecular ABMR gene transcripts for allograft attrition within patients with Banff classified ABMR [103]. The INTERCOMEX study has shown the added clinical value of these molecular gene expression transcripts for identifying rejection [104]. Two studies on molecular gene transcript classifiers further show that DSA-positive patients who present with high levels of these classifiers but show no histopathologic evidence of ABMR at light microscopy have more risk to develop histologic ABMR at subsequent biopsies compared to patients with low molecular ABMR gene transcript levels [105, 106]. Molecular analysis may thus offer additional prognostic value in case of a microscopically negative index biopsy, however these techniques are not yet available in most centers and require further validation. Some clinicians could perhaps consequently conclude that upon dnDSA detection, a certain amount of time should elapse before a biopsy is conducted, as this may decrease the chance of a negative biopsy result in those patients with molecular but not yet microscopic histological rejection. However, this may negatively impact potential efficacy of treatment in patients who would have more chronicity on later biopsies. Additionally, aforementioned studies on biopsy results upon subclinical dnDSA detection clearly show rejection in approximately 50% of cases. These would all be detected later by postponing a biopsy. An alternative strategy could entail a follow-up biopsy, if the index biopsy is negative. No study has been conducted which specifically addresses and compares the impact of these strategies on transplant outcomes. Therefore, more research on the optimal time of biopsy in patients with subclinical DSA is needed. Nevertheless, the additional prognostic value of renal biopsy information on both the Banff recognized rejection diagnosis and of the severity of the pathological lesions in patients with subclinical DSA seems clear. Therefore, despite the mostly single-center observational evidence, we strongly recommend to perform an allograft biopsy to further determine the pathogenicity and impact of developed subclinical dnDSA, if prognostication is desired.

### Efforts Should be Made to Standardize Testing and Reporting of DSA, Including Information on MFI, Their Plausibility and Possible Cross-Reactive Antigens/Epitopes (1B)

A prerequisite for any screening strategy is the availability of a suitable test system which is acceptable to the population and with facilities available for diagnosis and treatment. The SAB-SPI test is currently the test system of choice to define DSA. This method is semiquantitative, highly specific, sensitive and able to detect and identify anti-HLA antibodies. However, differences within and between laboratories impair reproducibility when it comes to the definition of DSA in both clinical practice and trials. A recent systematic review showed that the reporting of DSA in clinical trials had huge variability concerning assay type, DSA verification, MFI cutoff to define DSA and the prevention of prozone [107]. The level of “not reported” was determined at +/−15% for assay type, >30% for DSA verification and MFI cut off and around 80% for prozone treatment. Not only antibody tests have to be taken into account. Senev et al. [108] showed that 23% of DSA defined on a low resolution level could not be confirmed if correlated to second field high resolution HLA-typing results. Laboratory factors, as well as donor and patient factors, are inherent limitations to the testing and reporting of DSA [2, 3]. MFI values underestimate broad reacting specificities as Bw4/Bw6 or beads saturated with antibody. MFI does not reflect titer and one should bear in mind that SAB-SPI tests are qualitative, at best semiquantitative tests [42, 109]. Potential pitfalls notwithstanding, HLA-antibody detection and antigen/epitope specificity identification have never been as good as today. HLA-antibody assessment using solid phase assays including all major HLA-loci are already recommended in the 2017 North-American Sensitization in Transplantation: Assessment of Risk workgroup (STAR) report [109]. Initiatives such as the STAR workgroup [109, 110] are essential to clarify the expectations and limitations of current clinically used DSA detection methods. Clinicians need to receive comprehensive reports in a timely manner while being informed on the limitations of individual assays and results. Additionally, HLA-immunologists need to understand the clinical course of a patient after transplantation. Whereas HLA-labs are highly involved in the definition of acceptable antigens pretransplant, they are less involved in the posttransplant follow-up of individual patients. To increase clinical utility and validity, feedback should not only go from the lab to the clinic but also *vice versa*, resulting in both standardized analytical and clinical reporting. The following information can be helpful in DSA reporting: risk category of the patient at the moment of transplantation, DSA chronology, and the indication of DSA testing. This interaction is specifically needed to address the potential pitfalls of DSA screening in the entity of DSA-negative ABMR.

International standards for HLA-labs should focus on the different aspects that can interfere with the definition of DSA as a follow-up biomarker for subclinical DSA. These can include definition of MFI (Median, Mean, trimmed mean), signal-to-background calculation or plausibility evaluation.

Although the current SAB-SPI allow identification of DSA, further research is required to standardize DSA monitoring in patients with functioning grafts. The use of SAB-SPI methods measuring C3d or C1q complement fixing of DSA can have additional value but needs further validation and cannot currently be recommended as a biomarker for subclinical DSA monitoring, as conflicting retrospective studies exist [111, 112]. Conflicting studies also exist in regards to IgG subclass differentiation [55, 113]. The role of non-HLA post-transplant does not seem to be impactful, but the number of studies is currently limited [114].

Methods to detect B cell memory [115, 116] or to detect specific antibody parameters as affinity and avidity [117] are currently not available on a large scale nor are they ready as posttransplant monitoring biomarker. Further research on these topics is required.

### Whilst Post-Transplant Monitoring of Preformed DSA in Patients With Stable Graft Function Might be Helpful, Additional Clinical and Laboratory Parameters Should Also be Considered When Deciding if a Biopsy Should be Performed. (2C)

Development of dnDSA could prompt clinicians to further investigate a patient for underlying pathology. Here, we consider monitoring patients with subclinical preformed DSA. We will not argue against the validity and prognostic value of a biopsy *per se* in these patients. However, it is more difficult to determine a prompt to biopsy in patients with preformed DSA. It could be argued that post-transplant persistence of preformed DSA could prompt a biopsy as some preformed antibodies may gradually disappear from the circulation. Previous studies indicate that persistence of preformed DSA infers a higher risk of allograft loss and rejection than DSA that have disappeared [118–123], though some contradict this conclusion [88, 124]. Additionally, studies comparing allograft loss in patients with cleared preformed DSA versus no preformed DSA give conflicting results [118, 120, 125]. Furthermore, no study has examined the predictive value of clearance of preformed DSA. Thus, it is currently uncertain whether grafts in patients with cleared preformed DSA have a survival disadvantage or suffer higher rates of rejection compared to grafts in regular non-sensitized patients. It is therefore uncertain if clearance of preformed DSA should preclude a biopsy in patients without graft dysfunction. There is currently little evidence that post-transplant change in MFI of preformed DSA in patients with stable grafts has any predictive value. Early rise in preformed DSA MFI was associated with ABMR development in older studies [126, 127]. However, more recent in-depth analysis by Philpott et al. [128] of post-transplant temporal evolution of DSA indicated that allograft survival was impacted by the speed of change in MFI, rather than eventual delta MFI during the first month. They showed that patients with modulating preformed DSA (i.e., a rise then subsequent fall of MFI) had significantly better allograft survival than patients with sustained levels of preformed DSA (i.e., rising MFI and followed by sustained or stable MFI). This would indicate that a random point measurement of DSA MFI level in the early post-transplant course would provide minimal predictive information. Preformed DSA with high delta MFI compared to pretransplant levels could still be DSA which is undergoing a modulating course, which appears to infer less risk than DSA which had a more stable course in MFI. In this study, biopsies were only performed in case of allograft dysfunction, so it is difficult to extrapolate these results to patients with stable graft function. Moreover, delta MFI should be interpreted with caution in the absence of other clinical parameters, considering that the inter-laboratory variation of MFI can be as high as 62% [129] Consensus guidelines of the STAR workgroup are in line with this notion, as they state that any increase of MFI less than 50% is likely to be meaningless in otherwise “relaxed” situations [109]. Furthermore, even if the results of Philpott et al. [128] could be extrapolated to subclinical patients, they would only support careful monitoring in the first month post-transplant, as allograft survival was dependent on the evolution of DSA in that month. Unfortunately, no studies analyzed associations between late evolution in preformed DSA MFI and transplant outcomes. This leads to the conclusion that, although patients with preformed DSA and stable grafts can have latent rejection, there is currently no evidence to support the notion that monitoring these DSA alone provides a prompt to initiate further investigation of the patient. Additional clinical and laboratory parameters should thus also be considered, before deciding upon a biopsy in patients with preformed DSA. The lack of robust evidence regarding this topic is reflected in the grading of this recommendation. Alternatively, these patients might benefit from strategies utilizing protocol biopsies [2, 130] or a combined screening strategy using additional non-invasive biomarkers of rejection. A separate workgroup within the TLJ3.0 platform will publish consensus statements on the clinical validity and utility of these biomarkers and these methods are therefore beyond the scope of this consensus report.

### DSA MFI Levels or Complement Binding Ability (C1q, C4d, C3d) Should Not Influence Decision-Making Regarding Whether a Biopsy in Patients With Subclinical dnDSA Should be Performed. (2C)

Development of subclinical dnDSA may prompt further investigation of the patient, though it would be of interest to define other factors that would help stratify the risk of underlying graft pathology. This may prevent needless allograft biopsies in patients with subclinical dnDSA, considering that not all patients with dnDSA have recognizable ongoing ABMR at biopsy. Previous studies have shown that patients with ABMR more often have antibodies aimed at HLA class II, however this is also likely related to class II antibodies being the most commonly formed type. [22, 92, 131] Moreover, a recent large cohort study did not find any difference in the proportion of patients with HLA class I dnDSA who have underlying ABMR, as compared to class II dnDSA. [29] Additionally, dnDSA HLA-class specificity does not seem to be significantly associated with graft survival in multivariate analysis. [29, 131] This indicates there is not enough evidence to state that DSA HLA-class significantly attenuates the risk of a rejection diagnosis or the graft prognosis and therefore should not influence the decision to omit a biopsy in patients with subclinical dnDSA. Multiple studies have associated other DSA characteristics with worse outcomes, such as MFI level (sum of all DSA MFI or highest individual MFI) [22, 88, 132–136], certain IgG subclasses [55, 137, 138], or complement binding ability (C1q, C4d, C3d) [54]. However, most studies do not provide information on the negative predictive value of these characteristics, which would be the parameter of interest in deciding on whether to omit a biopsy. Prospective randomized studies are lacking and only a few studies investigated the predictive value of these DSA characteristics. Eskandary et al. [82] retrospectively studied 86 patients with subclinical DSA and associated highest MFI, sum of MFI and complement binding ability with underlying ABMR. However, the individual C-statistics were moderate at best for each characteristic (0.77, 0.75 and 0.65 respectively). Additionally, a combined model of maximum or sum of MFI and either C1q, C4d or C3d-positivity did not improve the predictive power of the base model of only MFI significantly. The authors found that while a higher MFI cutoff of >5000 or >10000 enjoyed a higher specificity for ABMR (0.86 and 0.99 for both MFI characteristics), the sensitivity drastically reduced from 0.82, 0,84 to 0.34, 0.43 and 0.30, 0.27 respectively. These MFI cutoffs subsequently result in low negative predictive value for ABMR in patients with subclinical dnDSA (MFI > 5000: 0.63, 0.67; MFI > 10000: 0.64, 0.65, for maximum MFI and sum of MFI, respectively). This indicates at least 30% of underlying ABMR would be missed by preclusion of a biopsy based on MFI cutoffs >5000 in subclinical patients. The fact that MFI values do not reflect the strength of the antibody titer might be an important cause of the poor correlation between MFI values and outcome [42, 109]. A recent study could not identify a relationship between MFI at first occurrence and outcome, only a profound >50% reduction of dnDSA MFI values was associated with better graft survival in a multivariate model [29]. Another study by Viglietti et al. [139] performed analyses with allograft loss as outcome in 186 patients with both subclinical and clinical DSA. They found an equally moderate C-statistic regarding maximum MFI in the total group of patients with post-transplant DSA (0.72). This was only marginally better in specifically dnDSA-positive patients (0.75). No analysis regarding specific MFI cut-offs was performed. While C1q-binding was found to significantly increase the fit of the base model, the numerical increase in C-statistic was a marginal 0.028 in dnDSA-positive patients (0.751–0.779) Interestingly, IgG3-positivity strongly increased the fit of the model with improvement of the C-statistic from 0.75 to 0.88. Yet this specific characteristic was predominately present in patients whose dnDSA were detected after development of allograft dysfunction. Only 2% of patients whose dnDSA were detected as a part of regular annual screening were IgG3-positive, yet 74% and 57% of these patients had ABMR at biopsy one and two years post-transplant respectively. These studies indicate that while some DSA characteristics such as higher MFI or IgG3 positivity might increase the likelihood of underlying pathology in dnDSA-positive patients with stable grafts, absence of these characteristics also definitely do not exclude it. Therefore, as robust supporting evidence is lacking, it seems that none of these studied DSA characteristics can be used reliably to preclude a biopsy in patients with subclinical DSA. We therefore currently do not recommend utilizing these DSA characteristics as an aid in deciding if a biopsy of patients with subclinical dnDSA should be performed.

### We Recommend Optimization of Maintenance Therapy, Including Addressing Non-Adherence in Patients Who Develop Subclinical dnDSA. Additional Treatment Should Only be Considered After Performing an Allograft Biopsy. (1C)

#### Optimization of Maintenance Therapy

A crucial element of a screening program is whether proper treatment exists and whether there is consensus on whom to treat. Optimization of maintenance therapy, which includes promoting adherence, reducing exposure to secondary risk factors such as hypertension and maintaining appropriate calcineurin inhibitor trough levels, has been recommended in previous consensus statements for the treatment of ABMR and TCMR [15]. Moreover, the consensus on managing modifiable risk in transplantation (COMMIT) workgroup addressed non-adherence and underexposure to immunosuppression as pivotal risk factors for poor transplant outcomes [140]. The importance of adequate exposure has also previously been demonstrated in patients with DSA. Multiple studies showed that DSA-positive patients with adequate exposure have better graft survival compared to DSA-positive patients who remain non-adherent or with iatrogenic underexposure to immunosuppression [22–24]. Development of dnDSA has been heavily correlated to underexposure to immunosuppression [22–28]. This risk factor for poor transplant outcomes can be addressed and this could be done irrespective of underlying histology, because dnDSA may still signal underexposure even if there is no microscopically visible rejection. However, the recently published OuTSMART trial, which analyzed the effects of optimization of maintenance therapy based on DSA monitoring results, seems to contradict these previous retrospective studies [21]. No significant difference was found in regards to graft survival between standard of care and optimization of maintenance therapy based on DSA monitoring. This randomized controlled trial (RCT) is qualitatively better evidence than observational research. However, it should be noted that the consenting participants in OuTSMART were already highly adherent at baseline. This is reflected by the low dnDSA incidence rate of 1.6% per year and may relate to the possibility of healthy survivor bias due to cross-sectional inclusion. Even though adherence improved significantly to even greater levels, it is uncertain whether it was to be expected that this should have resulted in improved graft survival. Nonetheless, this study does appear to show that broadening the immunosuppressive regimen does not have the expected effect on graft survival. Even the sensitivity analysis, which only included patients who were optimized to a triple therapy regime upon detection of dnDSA could not demonstrate survival benefit, though the confidence interval included both estimates of highly protective as well as highly hazardous effects. This could have been related to less allograft failure in DSA-positive patients than initially expected. Interestingly, total amount of biopsy-proven rejections was significantly lower in patients in the intervention arm, indicating that increased exposure does have immunological effect. Perhaps more benefit could be demonstrated if optimization of maintenance therapy is accompanied with biopsy-guided anti-rejection treatments as subclinical rejection was likely present in only 50% of subjects. More research in terms of broadening immunosuppressive regimen as a means of optimization of maintenance therapy is thus required for this to be recommended. Nevertheless, addressing non-adherence and secondary risk factors for progression are still important aspects of treatment, which we still strongly recommend in case of development of subclinical dnDSA. The ultimate goal is to optimize graft survival which includes taking into account competing mortality risk from infections, malignancies, and other toxicities.

#### Maintenance Immunosuppressive Target Levels

When subclinical donor-specific antibodies emerge, it becomes crucial to detect potential non-adherence and optimize the maintenance immunosuppressive regimen, unless there are contraindications present. In case of signs of ongoing alloimmunity, the convention in many center is often to switch to triple therapy with tacrolimus, mycophenolate analogues and maintenance steroids balanced against toxic side effects. Unfortunately, there is a current lack of strong evidence for exposure targets in kidney transplant recipients with subclinical dnDSA. To give some clinical directions, target tacrolimus exposure could be extrapolated from trough levels to prevent (additional) DSA [25, 28] rejection [141], and to improve graft survival [142–144]. Collectively, these studies suggest that maintaining the tacrolimus trough level between 5 and 8 ng/mL, which is in line with international recommendations, might prevent alloimmunity and optimize survival, albeit two studies suggested a potential lower threshold of 4 ng/mL in patients with very low intrapatient variability [141, 142]. However, whether this target range is helpful once caABMR ensues remains unknown. A study by Sablik et al. [145] did not find any survival difference between a tacrolimus trough greater or lesser than 5.9 ng/mL. Interestingly, they did find that higher intrapatient variability was significantly associated with poorer survival in patients with caABMR, suggesting that adherence and time in therapeutic range are probably more important exposure variables than attained trough levels within current clinical practice. Even less evidence is available regarding optimal mycophenolate exposure. A small single study found a trough >1.3 mg/L to prevent DSA formation [146]. It is reasonable to hypothesize that a clear exposure-relationship curve between mycophenolate and antibody formation might exist, considering the almost linear relation between MPA exposure and SARS-CoV-2 antibody formation [147]. No evidence is available for reintroduction of low-dose steroids, it is however often assumed that the anti-inflammatory effects and the diminished chance of acute rejection from maintenance steroids might have beneficial effects in the long-term but need careful balancing against side-effects [148]. Some evidence has emerged regarding the effectiveness of conversion from a CNI based immunosuppressive regime to costimulation blockade with belatacept [149]. Perhaps optimization of maintenance therapy could entail such a strategy, as it would effectively eliminate occult non-adherence due to the necessity of intravenous administration. Additionally, belatacept’s immunological mode of action may be more fitted for patients who have already developed a dnDSA as it interrupts T-follicular helper cell—B-cell interaction and could thus decrease B-cell stimulation and further reduce the evolution of DSA formation [29, 150]. Some studies have shown effectiveness of belatacept on DSA levels and on the (lower) incidence of ABMR in sensitized patients [149–152]. Interestingly, DSA positivity was not associated with graft loss in a small cohort of patients converted to belatacept, though the presence of aABMR with MVI was independently associated with treatment failure [153]. It has to be noted however, that the incidence of TCMR was significantly increased, especially in patients converted within the first year post-transplant [154]. We therefore recommend more research to be conducted on the role of costimulation blockade as a means to optimize maintenance therapy in patients with subclinical DSA.

#### Pre-Emptive Treatment *In Lieu* of an Allograft Biopsy

In regards to further treatment of patients with subclinical dnDSA before conducting a biopsy, evidence is lacking. Only one small cohort study has been identified, in which patients with subclinical DSA were treated with bortezomib, PP, IVIG and corticosteroids without performing a biopsy to confirm rejection [155]. This study showed that patients who achieved DSA clearance had more stable 2 year allograft function compared to those with persistent DSA. However, no control group was included and thus it cannot be concluded that improvement in outcome was due to treatment. Furthermore, irrespective of efficacy, subjecting all patients with subclinical dnDSA to such a strong and broadly targeting immunosuppressive regimen might be difficult to justify, considering that roughly half of this population have no underlying observable histological injury [30, 57, 78, 80–83]. In addition, transient spontaneous negativity of dnDSA has been observed in 24% of patients with subclinical dnDSA and complete clearance of dnDSA has been observed in around 10% of patients [29]. Lastly, identification of the Banff classified type of rejection through a biopsy will ensure that patients with underlying cell-mediated rejection are not unnecessarily subjected to therapy aimed at antibodies and *vice versa*. We therefore do not recommend additional preemptive treatment of patients with subclinical dnDSA, besides optimization of maintenance therapy, without performing an additional allograft biopsy.

#### Treatment of Subclinical T-cell Mediated Rejection

Amongst dnDSA-positive patients with underlying rejection, those with subclinical TCMR may have the best evidence for gained benefit. Treatment of subclinical TCMR has been investigated in multiple studies (Table 5). A literature review by Mehta et al. [156] revealed that most available studies [157–161] at the time showed that subclinical acute TCMR (aTCMR) is associated with inferior outcomes. Choi et al. [162] observed significantly lower 10 years allograft survival in patients with untreated early subclinical TCMR vs. non-rejectors (62.3% vs. 96.2%). Consequently, ESOT advocates subclinical aTCMR to be considered as primary efficacy endpoints in clinical trials [163]. The first evidence of treatment came from a randomized trial by Rush et al. [161]. They showed that treatment of early subclinical TCMR detected in protocol biopsies leads to lower chronicity scores, less late rejections and more stable and lower creatinine levels at 2 years post-transplant than untreated patients. Another RCT by Kurtkoti et al. [160] showed similar results in regards to lower creatinine levels at 6 and 12 months. These older studies could be criticized for having been conducted before the tacrolimus era and thus being less applicable to current practice. A more recent randomized trial of early protocol biopsy and treatment of subclinical TCMR in patients with tacrolimus and mycophenolate analogues showed no benefit of treatment [164]. There was no difference in renal function at 6 months and chronic histology scores were in fact higher in the treatment arm. This study was, however, limited by the relatively low frequency of subclinical rejection at early protocol biopsy, as only 4.6% showed subclinical TCMR. Additionally, chronicity scores in the control arm appeared to improve from implantation to the 6 months biopsy in some patients with seemingly no additional intervention. This perhaps indicates other unknown factors may have influenced the results of this study and limits the potential conclusions that can be drawn from it. In terms of more recent observational research, Seifert et al. [165] analyzed protocol biopsies at 3 and/or 6 months in 120 pediatric patients. They showed that 13 treated patients with subclinical aTCMR still had a significantly increased risk of meeting the composite endpoint of death-censored allograft loss and acute rejection at 5 years post-transplant, compared to patients without rejection. However, choice of treatment modality of this low number of patients was at the discretion of the physician. In contrast, larger recent studies showed no significant difference in delta creatinine, odds of 50% eGFR loss, or allograft survival between subclinical TCMR patients treated standardly with pulse steroids and a control group without TCMR at protocol biopsy; [57, 166]. It should be noted that these studies were mainly performed in DSA-negative patients. Thus, less is known about treatment of DSA-positive subclinical TCMR cases, although there is a broad consensus about the detrimental long-term consequences on ongoing inflammation in renal allografts [163]. However, Cherukuri et al; [24] analyzed the effect of treatment with steroid pulses on patients with TCMR and/or DSA, although these were not specifically subclinical cases. Patients with underlying TCMR and no DSA had no significant risk of graft loss. However, TCMR with concurrent DSA was a significant risk factor for 4 years allograft attrition in multivariate analysis, even when treated. Crucially, this significant risk was attributable to non-adherence. Adherent and pulse steroid treated patients with DSA and TCMR had no increased risk of allograft loss compared to patients without DSA and rejection, whereas non-adherent, pulse steroid treated patients with DSA and TCMR had drastically lower graft survival rates. This seemingly indicates that DSA-positive patients with underlying TCMR may still be amendable to current treatment modalities, provided they are adherent. This further signals that strengthening adherence is an important treatment option and is recommended by us and others in patients with dnDSA [15, 140]. There are currently no guidelines on the treatment of subclinical TCMR [163]. A recent systematic review and meta-analysis by Ho et al. [167] showed through the included retrospective studies that most centers seem to treat subclinical TCMR (Banff 1a or higher) with pulse steroids and occasionally thymoglobulin. This is in line with two recent surveys, which show that more than 90% of North-American transplant centers have implemented pulse steroids or lymphocyte depleting antibodies as standard of care in these patients [168, 169]. Currently, ESOT is surveying this in Europe as well.

**TABLE 5:** Summary of studies on outcome of treated and untreated subclinical.

Study	Type of study	Total patients (n)	Time of biopsy	Total subclinical TCMR (n) or (%)	Treatment of subclinical TCMR	Outcome
Nankivell et al. [157]	Retrospective Single center	961	1, 2 weeks 1, 3, 6, 12 months post-transplant Annually thereafter	6.9% of all biopsies TCMR	Methylprednisolone in 22.9% of TCMR and 12.3% of B-TCMR	Biopsies taken >3 months post-transplant with subclinical TCMR associated with higher ci and ct scores at 1 year biopsy
				23.4% of all biopsies B-TCMR		Persistent TCMR associated with more significant decline in eGFR at 2 years
Moreso et al. [158 ]	Retrospective Single center	372	Protocol biopsy during initial 6 months post-transplant “For cause”	74 subclinical TCMR	None	15 years DCGS lower in patients with CAN + TCMR compared to no rejection RR 1.86 (1.11–3.12)
				65 subclinical TCMR + CAN		
Scholten et al. [159]	RCT	126 1:1 TAC vs. CsA	Protocol biopsy at 6 and 12 months post-transplant	At 6 months: 7.4% TCMR and 23.4% B-TCMR	None	Less subclinical TCMR in TAC group
			At graft dysfunction	At 12 months 14.3% TCMR 24.5% B-TCMR		Subclinical TCMR not associated with creatinin clearance at 2 years
Kurtkoti et al. [160]	RCT	102	Protocol biopsy at 1, 3 months post-transplant vs. Indication only	Protocol biopsy group at 1, 3 months: 17.3%, 12%	Pulse steroids	Serum creatinin significantly higher at 6 and 12 months in control group vs. protocol biopsy group
		1:1				At 6 months: 137 ± 35 μmol vs. 113 ± 29 μmol (*p* < 0.001)
		Protocol biopy vs. Only indication biopsy				At 12 months: 134 ± 36 μmol vs. 106 ± 29 μmol (*p* < 0.001)
Rush et al. (1998) [161]	RCT	72	Protocol biopsy at 1, 2, 3, 6, 12 months vs. Protocol biopsy at 6, 12 months	In early biopsy group: Subclinical TCMR at 1, 2, 3, 6 months: 43%, 32%, 27%, 15%	Pulse steroids	Significantly higher amount of patients with ci + ct scores ≥2 in control group vs. early biopsy group 24% vs. 6% at 6 months (*p* < 0.04)
		1:1		In late biopsy group: Subclinical TCMR at 6 months: 32%		Significantly higher creatinin at 2 years in control group vs. early biopsy group 183 ± 22 μmol/L vs. 133 ± 14 μmol/L (*p* < 0.05)
		early biopsies vs. later biopsy				
Choi et al. [162]	Retrospective Single center	304	Day 14 Post-transplant	40	None	10 years graft survival subclinical TCMR vs. no rejection: 62.3% vs. 96.2% (*p* < 0.05)
Rush et al. (2007) [164]	RCT	218	Protocol biopsy at 1, 2, 3, 6 months vs. Protocol biopsy at 6months	In early biopsy group: Subclinical TCMR at 1, 2, 3, 6 months: 5.7%, 0%, 8.1%, 8.9%	Pulse steroids	Significantly higher increase in ci + ct scores ≥2 at 6 months compared to baseline in early biopsy/treatment group vs. control group (1.12 ± 1.36 and 0.57 ± 1.02, *p* = 0.04)
		1:1		In late biopsy group: Subclinical TCMR at 6 months: 6.0%		No significant difference in creatinin clearance or proteinuria at 6 months between groups
		early (<6 months) biopsies/treatmentvs no biopsy				
Loupy et al. [57]	Retrospective Single center + External validation	1,001	Protocol biopsy at 1 year	132	Pulse steroids	No significant difference in 8 years allograft survival or 8 years eGFR between subclinical TCMR vs. no rejection
Seifert et al. [165]	Retrospective Single center	103	Protocol biopsy at 3, 6 months	37	Increased maintenance immunosuppression, pulse steroids or thymoglobulin at discretion of physician	Significantly higher 5 years freedom from composite endpoint of acute clinical rejection or allograft loss in no rejection vs. untreated subclinical B-TCMR (*p* < 0.001)
						No significant difference in 5 years composite endpoint between treated subclinical B-TCMR vs. no rejection
						Significantly higher 5 years composite endpoint in no rejection vs. treated subclinical TCMR
Hoffman et al. [166]	Retrospective Single center	192	Protocol biopsy at 3, 12 months	56	Pulse steroids (Banff 1A/B) or thymoglobulin (Banff ≥ 2A)	No significant difference in delta creatinin between 3 and 24 months or odds of 50% decline in eGFR between 3 months and final follow up between subclinical TCMR vs. no rejection

TCMR CAN, Chronic allograft nephropathy; ci, Interstitial fibrosis; ct, Tubular atrophy; CsA, Ciclosporin; DCGS, Death-censored graft survival; RCT, Randomized controlled trial; TAC, Tacrolimus; TCMR, T-cell mediated rejection; B-TCMR, Borderline TCMR.

#### Treatment of Subclinical Antibody-Mediated Rejection

As a substantial amount (40%–50%) of patients with subclinical dnDSA will have signs of ABMR upon biopsy, it is important to review the evidence for treatment options in these patients. Recent consensus guidelines concluded that there is very little evidence for efficacy of current treatment protocols for ABMR in patients with dnDSA [15]. However, a retrospective study showed an incremental improvement in the treatment of ABMR; [170]. In addition, a small phase II prospective randomized trial with an IL-6 inhibitor has shown some promising results in chronic active ABMR (caABMR), and is currently being studied in a large multicenter phase III RCT [171, 172]. Additional evidence is emerging on the effectiveness of costimulation blockade, as discussed above, and anti-CD38 therapy in patients with aABMR and caABMR, the latter of which is currently being investigated in a phase II RCT in the form of felzartamab [149, 173]. In light of emerging data one may conclude that (early) acute ABMR with dnDSA (but without transplant glomerulopathy) could be more responsive to maintenance treatment optimization as well as PP and IVIG and eventually novel treatment regimens than patients with caABMR or cABMR, albeit all the treatment options have a low amount of supporting evidence. Active research in this area is ongoing and ABMR definition is becoming more precise [174]. Thus, there could potentially be benefit in finding and treating patients with early (subclinical) forms of ABMR before they present late with irreversible chronic lesions and clinical dysfunction. Some retrospective studies seem to support this hypothesis (Table 6). Parajuli et al. [102] showed similarly good post-biopsy allograft survival in patients with subclinical ABMR treated with IVIG and PP, as compared to protocol biopsied dnDSA-positive patients without rejection. Additionally, patients with treated subclinical ABMR had significantly better allograft survival than DSA-negative patients with indication biopsies or patients with treated clinical ABMR. Importantly, there was no difference in outcome between subclinical ABMR based on preformed DSA (type 1) vs. dnDSA (type 2). However, it must be noted that the post-biopsy follow-up time in patients with subclinical ABMR was relatively low at 31.0 ± 15.8 months. Orandi et al. [175] showed that patients with mostly type 1 subclinical ABMR treated by PP and in some situations rituximab or eculizumab had no significantly different rate of 5 years death-censored allograft loss compared to ABMR negative matched controls, whereas untreated patients had significantly more 5 years death-censored graft attrition rates compared to their control group. In addition, Yamamoto et al [79]. described some beneficial effects of PP and rituximab in 8 out of 18 (44%) of patients with subclinical type 2 ABMR whereby DSA levels reduced significantly or histological injury stabilized upon rebiopsy. In contrast, studies by Bertrand et al. [77] and Loupy et al. [57] found that allograft survival in treated subclinical ABMR patients was still significantly worse than patients without rejection. However, only 39% of patients with subclinical ABMR in the study by Loupy et al. [57] received specific treatment for subclinical ABMR and no analysis was performed comparing the treated and untreated group. It is apparent that more robust research on the effectiveness of treatment of subclinical ABMR is warranted. Nonetheless, the overall risk-benefit balance seems to be in favor of screening of DSA, which could result in early optimization of maintenance therapy. Moreover, further biopsy-guided treatment of subclinical TCMR and subclinical ABMR may be more effective than later treatment of clinical rejections, though evidence for this notion is more limited, as reflected in the grading of this recommendation.

**TABLE 6:** Summary of studies on outcome of subclinical ABMR with or without treatment.

Study	Type of study	Total patients (n)	Total subclinical ABMR (n) or (%)	Type 1 or type 2 ABMR	Time of biopsy	Treatment of subclinical ABMR	Outcome
Parajuli et al. [102]	Retrospective single center	220	25 (all treated)	Type 1 and 2	Detection of dnDSA	≤3 months post-transplant: Pulse steroids, IVIG, PP	No significant difference in 5 years post-biopsy DCGS between treated subclinical ABMR and no rejection
					Protocol biopsies in case of pretranplant DSA		Significantly better 5 years post-biopsy DCGS in treated subclinical ABMR than clinical ABMR and than DSA- indication biopsies
					50% rise in MFI	≥3 months post-transplant: Pulse steroids, IVIG, situationally RTX	(92% vs. 54%, proportion of DSA- indication biopsies with DCGS not provided)
					Graft dysfunction		No significant difference in post-biopsy DCGS between type 1 or type 2 subclinical ABMR.
Orandi et al. [175]	Retrospective single center	2097	77 (41 treated)	Uncertain Mostly type 1	Protocol biopsies at 1,3,6, 12 months post-transplant in HLA or ABOi incompatible transplants	PP + Situationally RTX or eculizumab	No significant difference in DCGS between treated subclinical ABMR and ABMR free matched controls. HR 1.73; 95% CI: 0.73–4.05; *p* = 0.21
							Significantly worse DCGS in untreated subclinical ABMR vs. ABMR free matched controls. HR 3.34; 95% CI: 1.37–8.11; *p* = 0.008
Yamamoto et al. [79]	Retrospective single center	43	18 (all treated)	Type 2	At dnDSA detection	Plasmapheresis and RTX	Significant decrease of MFI in 6 out of 18 patients
							Within 10 patients with rebiopsy, 4 had improvement or no change in graft histology
Bertrand et al. [77]	Retrospective Multicenter	123	51 (19 treated)	Type 2	At dnDSA detection	A combination of IVIG/PP/RTX	Significantly worse 8 years biopsy DCGS in subclinical ABMR patients vs. no rejection. (78% vs. 97%, *p* < 0.01)
							No significant difference in 8 years post-biopsy DCGS between treated and untreated subclinical ABMR
Loupy et al. [57]	Retrospective single center + External validation	1,001	142 (56 treated)	Type 1 and 2	Protocol biopsy at 1 year post-transplant	IVIG, PP, RTX	Significantly worse 8 years graft survival probability in subclinical ABMR vs. no rejection (56% vs. 90%, *p* < 0.0001
							Significantly faster decline of eGFR over 8 years in subclinical ABMR vs. no rejection (p not provided)
							No analysis in regards to treated vs. untreated subclinical ABMR

ABMR, Antibody-mediated rejection; DCGS, Death-censored graft survival; DSA, Donor-specific antibody; dnDSA, *de novo* DSA; eGFR, Estimated glomerular filtration rate; IVIG, Intravenous immunoglobulins; MFI, Mean fluorescence intensity; PP, Plasmapheresis; RTX, Rituximab.

### Cost-Effectiveness of DSA Monitoring in Patients With Stable Graft Function Will Depend on Incidence Rate of dnDSA and Importantly on Size Effect of Treatment (2D)

Assessment of the balance between medical risks and benefits of early case finding may determine that a screening program is medically justified, though this assessment does not necessarily determine whether it is cost-effective. As transplant centers have finite resources, DSA screening should be economically balanced to the cost of medical expenditure as a whole. Important aspects are the costs of the screening test and of the consequences of a missed case. The costs of a patient with graft loss due to ABMR who proceeds to renal replacement therapy far exceed the costs of those who retain their transplant by over €40.000 per year [176]. If one assumes that graft losses to ABMR account for around 1/3 of all graft losses [17] and takes into consideration the costs and benefits of potential treatment as well as morbidity and mortality rates of those treatments, then DSA screening seems justifiable on first glance. Unfortunately, evidence in the literature on this topic is very scarce. Kiberd et al. [177] performed a DSA monitoring cost-effectiveness modelling study. They found that costs per increased quality-adjusted life year (QALY) could range from $127.000 to $444.000, depending on the estimated efficacy of treatment and on the incidence rate of dnDSA. However, the model did not account for the fact that costs saved by not screening and treating early would still partly be spent later on treating patients when they do present with clinical dysfunction. This means that the presented costs per QALY are likely an overestimation, especially considering that most of the projected costs were attributed to the treatment of found cases, instead of DSA screening itself. Nonetheless, the basis for a cost-effective screening strategy is adequately illustrated through this modelling example. The only real-world data regarding cost-effectiveness comes from the previously mentioned OuTSMART study [21]. The incidence rate of dnDSA in this study population was lower than expected at 1.6% per year. This, in combination with no found benefit of optimization of maintenance therapy, resulted in a staggering incremental cost-effectiveness ratio of £1.692.222 per QALY for monitoring for DSA. As stated before, development of dnDSA pertains to multiple risk factors, and particularly to the immunological risk and epitope mismatch [4, 28, 46, 178]. The varied reported incidence rate in current literature likely attests to this, as some report a steady rate ranging from 1.5% to 5.4% per year in immunological low-risk patients [22, 179–181]. Others report increased incidence in the first year ranging from 3.2% to even 20% with a lower steady yearly rate thereafter ranging from 0.8% to 4.3% [30, 182, 183]. The lower incidence rate in OuTSMART could thus perhaps be a reflection of better organ allocation, better post-transplant overall care or it could simply reflect a different population in terms of age, healthy survivor bias from cross-sectional inclusion, ethnicity or proclivity to adhere to their medication as compared to the populations in the mentioned reports in the literature. Nonetheless, the results of this trial provide real-world validation of the modeling study by Kiberd et al. [177], as it shows that cost-effectiveness of DSA monitoring is dependent on the incidence rate of dnDSA and effect of treatment. Whether or not DSA monitoring is cost-effective, may thus in fact differ between centers, as incidence rate, local treatment protocols, and allograft biopsy strategy in case of subclinical dnDSA may differ. More trials, with standardized DSA definition and reporting, in various populations with additional allograft biopsies in case of subclinical dnDSA are ultimately needed to fully determine the cost-effectiveness of DSA monitoring.

### Monitoring for dnDSA During Functional Graft Life Is a Continuous Process and Should Not Cease Upon Detection of dnDSA (2C)

Case-finding should be a continuing process and not a “once and for all” project. As new cases of subclinical rejection accumulate over time post-transplantation, DSA screening cannot be a one-time effort [29, 34]. The intensity and the longevity of the monitoring strategy should be reflected by the *a priori* chance of development of dnDSA over time. A recent large retrospective analysis shows that of 400 patients with dnDSA, 20% were found within the first year, 60% within 5 years and 85% within 10 years post-transplant, clearly indicating that even after 10 years post-transplant, patients may still develop dnDSA [29]. Unfortunately, as shown previously, the annual dnDSA incidence rate is not fully clear. Nevertheless, all studies indicate that dnDSA are constantly evolving and that the incidence does not reduce significantly after 1 year post-transplant. This subsequently implies that any time-limited monitoring strategy, although less costly, would be medically arbitrary and would miss new subclinical cases that occurred after screening ceased. The OuTSMART trial attests to this notion, as incidence rate did not diminish after a set amount of prospective monitoring years [21]. Another point of contention is whether monitoring should be continued for persistence or development of new dnDSA once a dnDSA has been detected. A retrospective study by DeVos et al. [184] found that patients with >60% positive DSA measurements in at least 3 separate assessments are more likely to progress to allograft loss than those with <60% positive measurements. López del Moral et al. [29] showed that dnDSA which eventually disappear, either temporarily or permanently, are associated with a lower rate of allograft loss than those who persist. Additionally, they showed that development of multiple dnDSA is associated with worse allograft survival, though this association was no longer statistically significant in multivariable analysis. In contrast, Kim et al. [88] found that resolved dnDSA was not associated with less decline in renal function. These studies, while somewhat conflicting, overall seem to suggest that newly developed dnDSA which eventually disappear are less likely to be associated with subsequent allograft loss. This implies that continued monitoring after dnDSA have already developed could serve important prognostic purposes. Moreover, additional dnDSA may develop, which could be cause for an additional allograft biopsy. Current low-grade evidence thus suggests that monitoring should not be discontinued after a set amount of post-transplant years, nor upon development of dnDSA.

### The Optimal dnDSA Monitoring Scheme has Not Been Established, but a Routine Approach Would Be Antibody Monitoring at Three to Six Months Post-transplant and Annually Thereafter. (2C)

Another dilemma in regards to the continuing process of case finding entails the intensity of monitoring. In an ideal world, development of dnDSA would be noted immediately. But this would require a frequency of monitoring that is unlikely to be feasible. Centers which perform routine DSA monitoring seem to do so annually with one or more additional measurements in the first year post-transplant [30, 77, 80]. A more personalized approach could be monitoring intensity based on the immunological risk, this may also be more cost-efficacious, as lower risk patients could be subjected to less frequent screening. Monitoring intensity stratification based on HLA-matching might be easy to establish. Naturally, recipients of a completely HLA-identical donor kidney have no risk of developing HLA-DSA. Completely HLA-identical transplants are, however, rare. Most DSA appear to be aimed at HLA-DQ [185], though López del Moral et al. [29] showed that the proportion of patients with a full HLA-DQ match who developed dnDSA was comparable to those with a full HLA-B or HLA-DR match. This indicates that other HLA-loci mismatches should not so easily be disregarded. More recent evidence regarding molecular eplet HLA mismatching has emerged, whereby a low DQ/DR eplet mismatch was found to carry a negligible risk for development of DQ or DR dnDSA [26, 28]. In addition, analysis of the predictive value of the PIRCHEII and HLA-matchmaker molecular eplet mismatch algorithms showed that low eplet mismatch was associated with reduced probability of dnDSA development for both class I and II HLA-loci [186]. Lastly, post-hoc analysis of the CELLIMIN trial showed that high molecular eplet mismatch load was associated with development of dnDSA for both HLA-classes [187]. These studies indicate that low levels of total eplet mismatch load could be a reason to lower DSA monitoring intensity or even omit it. Personalized DSA monitoring intensity based on molecular mismatch thus seems promising. However, further validation of this risk-stratification technique in prospective trials on DSA screening is needed and more research is thus recommended. Currently, no study has been conducted which compares outcomes of different monitoring frequency strategies. Notwithstanding, the study by Parajuli et al [102] shows that patients with subclinical dnDSA who are detected, biopsied and treated through a strategy consisting of screening after 6 months and annually thereafter have good outcome. This suggests that more intensive monitoring may be unnecessary. Additionally, a monitoring interval greater than 1 year might be ill-advised, as studies in untreated subclinical ABMR show more chronic lesions within 1 year post-diagnosis [72, 97]. This may indicate that patients detected beyond 1 year from inception of the dnDSA may be more difficult to treat. Lastly, considering multiple studies have indicated increased incidence of development of dnDSA in the first year post-transplant, it might be advisable to perform an additional measurement within three to 6 months post-transplant [29, 30, 182, 183]. It thus appears from current low-level evidence that, until more robust immunological risk-stratification methods are validated, monitoring strategies consisting of screening within the first three to 6 months post-transplant and annually thereafter may seem pragmatic. However, more prospective research is needed to determine the optimal monitoring strategy.

## Summary and Next Steps

The authors suggest that, based on current available evidence and the assessment of each individual Wilson & Jungner criterium, monitoring for development of dnDSA has clinical utility to further optimize long-term graft survival. A routine approach for such a strategy could be annual monitoring with an additional assessment within the first three to 6 months post-transplant. Monitoring should not cease after a certain amount of time or after dnDSA has already developed. Subclinical dnDSA development should lead to promotion of adherence and addressment of secondary risk factors. Further treatment should only be considered after performing an allograft biopsy to diagnose underlying rejection. Evidence for further treatment guided by such biopsies in subclinical patients is limited. However, certain patients with early rejection may respond to it empirically and treatment of subclinical TCMR has become standard of care in most centers. Novel treatments may provide additional efficacy in terms of prolonging allograft survival in the near future. Ultimately, further prospective trials are necessary to fully determine the benefits of such treatment strategies and their cost-effectiveness. Monitoring preformed DSA and their evolution in the subclinical setting post-transplantation with currently available validated assays may not provide a clear enough signal for possible underlying pathology. Additional clinical and laboratory parameters should therefore be considered before deciding to perform a biopsy in these patients. However, this does not preclude DSA monitoring in these patients, as development of additional dnDSA should equally lead to further investigation and treatment of these individuals.

## Data Availability

The original contributions presented in the study are included in the article/supplementary material, further inquiries can be directed to the corresponding author.

## Group Members of Workgroup Composition

The ESOT workgroup subclinical DSA monitoring comprised of all main authors, as well as the following panel of independent experts: David Cucchiari, Lucrezia Furian, Giuseppe Ietto, Georgios Liapis, Lorna Marson, Mikhail Nozdrin, Michele Rossini, Stella Stabouli, Jelena Stojanovic, Stathis Tsiakas, Oleksei Voroniak, Angeliki Vittoraki, Gianluigi Zaza.

## Abbreviations

aABMR, Active antibody mediated rejection; ABMR, Antibody mediated rejection; aTCMR, Acute T-cell mediated rejection; caABMR, Chronic active antibody mediated rejection; cABMR, Chronic antibody mediated rejection; CDC, Complement-dependent cytotoxicity assay; COMMIT, Consensus On Managing Modifiable risk In Transplantation workgroup; CTS, Collaborative Transplant Study; dnDSA, *de novo* donor-specific antibody; DSA, Donor-specific antibody; eGFR, Estimated glomerular filtration rate; ESOT, European Society for Organ Transplantation; GRADE, Grades of Recommendation Assessment, Development and Evaluation; HLA, Human leukocyte antigen; IgG, Immunoglobulin G; IVIG, Intravenous Immunoglobulins; KTR, Kidney transplant recipients; MFI, Mean fluorescence intensity; MHC, Major histocompatibility complex; MVI, Microvascular inflammation; PP, Plasmapheresis; TCMR, T-cell mediated rejection; TLJ, Transplantation Learning Journey; RCT, Randomized controlled trial; SPI, Solid phase immunoassay; STAR, North-American Sensitization in Transplantation: Assessment of Risk workgroup; Subclinical DSA, Donor-specific antibody that has been noted in patients who otherwise do not show any sign of clinical dysfunction of the allograft, such as significantly increased proteinuria or decreased eGFR; QALY, Quality-adjusted life year.
